# Prevalence of perinatal depression among Japanese women: a meta-analysis

**DOI:** 10.1186/s12991-020-00290-7

**Published:** 2020-06-26

**Authors:** Keita Tokumitsu, Norio Sugawara, Kazushi Maruo, Toshihito Suzuki, Kazutaka Shimoda, Norio Yasui-Furukori

**Affiliations:** 1 Department of Psychiatry, Dokkyo Medical University School of Medicine, Tochigi, 321-0293 Japan; 2 Department of Neuropsychiatry, Towada City Hospital, Towada, Japan; 3 Department of Clinical Epidemiology, Translational Medical Center, National Center of Neurology and Psychiatry, Kodaira, Japan; 4 Department of Biostatistics, Faculty of Medicine, University of Tsukuba, Ibaraki, Japan; 5 Department of Psychiatry, Juntendo Koshigaya Hospital, Saitama, Japan; 6 Department of Neuropsychiatry, Graduate School of Medicine, Hirosaki University, Hirosaki, Japan

**Keywords:** Perinatal depression, Prenatal depression, Postpartum depression

## Abstract

**Background:**

Perinatal depression is one of the important mental illnesses among women. However, not enough reviews have been done, and a certain consensus has not been obtained about the prevalence of perinatal depression among Japanese women. The purpose of our study is to reveal the reliable estimates about the prevalence of perinatal depression among Japanese women.

**Method:**

We searched two databases, PubMed and ICHUSHI, to identify studies published from January 1994 to December 2017 with data on the prevalence of antenatal or postnatal depression. Data were extracted from published reports.

**Results:**

We reviewed 1317 abstracts, retrieved 301 articles and included 123 studies. The point prevalence of postpartum depression at 1 month was 14.3% incorporating 108,431 Japanese women. The period prevalence of depression at pregnancy was 14.0% in the second trimester and 16.3% in the third trimester. The period prevalence of postpartum depression was 15.1% within the first month, 11.6% in 1–3 months, 11.5% in 3–6 months and 11.5% in 6–12 months after birth. We also identified that compared with multiparas, primiparas was significantly associated with a higher prevalence of postpartum depression; the adjusted relative risk was 1.76.

**Conclusions:**

The prevalence of postpartum depression at 1 month after childbirth was found to be 14.3% among Japanese women. During pregnancy, the prevalence of depression increases as childbirth approaches, and the prevalence of depression was found to decrease in the postpartum period over time. In addition, we found that the prevalence of postpartum depression in primiparas was higher than that in multiparas. Hence, we suggest that healthcare professionals need to pay more attention to primiparas than multiparas regarding postpartum depression.

## Background

Perinatal depression, a mental illness that occurs either during pregnancy or within the first 12 months after delivery, affects the health and development of mothers and children [1, 2]. In 1968, Pitt reported that the prevalence of postpartum depression was 11% [3]. Epidemiological investigations have been conducted worldwide since then. In 1987, Cox developed the Edinburgh Postnatal Depression Scale (EPDS) [4], and screening measures have since progressed rapidly. In 1996, in the first meta-analysis of postpartum depression, the prevalence of postpartum depression was reported to be 13% [5]. Recently, estimates of the prevalence of postpartum depression in Western countries have reportedly been in the range of 13–19% [6].

Postpartum depression has been reported to occur due to biological [7], psychological and social problems. Social support from family members has a strong impact on postpartum depression [5]. Since the establishment of an equal employment policy for women in 1985, the employment rate of women has rapidly increased in Japan. However, there is insufficient social infrastructure for childcare, such as daycare, and men are not very involved in parenting. In addition, with the aging population and the increasing prevalence of nuclear families, social support in the perinatal period tends to be insufficient. In particular, the aging rate is 27.3% [8], which is the highest rate among developed countries, and support from family members, such as maternal parents, is weakening. For this reason, mental stress in women during the perinatal period is strong, and the risk of developing depression may be high. Therefore, it is problematic to apply current epidemiology data from different countries and regions to the Japanese context because of the social differences. Previous reports have suggested that perinatal depression may be affected by differences in economic status, social support, or ethnicity in the country where patients live [2, 5]. For this reason, we thought it would be relevant to conduct research focused on the country and culture of Japan.

In recent years, a large, prospective nationwide cohort study (*n* = 82,489) called the “Japan Environment and Children’s Study” (JECS) showed a 13.7% prevalence of postpartum depression among women 1 month after childbirth [9]. Although other studies on postpartum depression with various sample sizes have been carried out in Japan, with most of them written in Japanese, meta-analyses have not been conducted. For this reason, we collected articles for this study including those written in Japanese. The aim of our meta-analysis was to calculate a reliable estimate of the prevalence of postpartum depression among Japanese women. In addition, some studies have reported that the birth experience influences postpartum depression [9, 10], while other studies have indicated that there is no relation between the childbirth experience and postpartum depression [11, 12]. Therefore, we undertook a subanalysis of the relationship between postpartum depression and the childbirth experience.

## Method

### Study selection

This systematic review was reported according to the Preferred Reporting Items for Systematic Reviews and Meta-Analyses (PRISMA) standards (a protocol used to evaluate systematic reviews) [13]. We searched for published studies related to perinatal depression in the PubMed electronic database. The search phrase was ((pregnancy [ALL] OR antenatal [ALL] OR prenatal [ALL] OR gestation [ALL] OR postnatal [ALL] OR postpartum [ALL] OR postpartal [ALL] OR perinatal [ALL] OR puerperium [ALL] OR puerperal [ALL] OR postbirth [ALL] OR post-birth [ALL]) AND (depression [ALL] OR depressive [ALL] OR mood disorder [ALL] OR affective disorder [ALL]) AND (Japan [ALL] OR Japanese [ALL])).

In addition, the ICHUSHI database (http://search.jamas.or.jp/) was searched for articles written in Japanese. ICHUSHI contains bibliographic citations and abstracts from biomedical journals and other serial publications published in Japan. We used comparable Japanese search terms without the terms “Japan” and “Japanese” to search ICHUSHI.

The two electronic databases, PubMed and ICHUSHI, were searched for studies published from January 1, 1994, to December 31, 2017. We excluded older literature before the release of the Diagnostic and Statistical Manual of Mental Disorders, Fourth Edition (DSM-IV) [14]. Then, we examined the list of references included in the articles.

### Inclusion and exclusion criteria

Studies were eligible for inclusion if they (a) included women who were 16 years or older; (b) assessed prenatal or postpartum depression using a validated self-report instrument; (c) reported the results of peer-reviewed research based on cross-sectional or prospective studies; and (d) reported data to estimate the prevalence of prenatal or postpartum depression using the EPDS or Center for Epidemiologic Studies Depression Scale (CES-D). Studies were excluded if they (a) recruited only high-risk women; (b) reported results for only a subsample of a study population; (c) reported duplicate data from a single database; (d) reported only mean data; (e) did not report a cutoff point for depression; or (f) had < 100 participants (these studies were excluded to avoid a small-study effect) [15]. For studies with duplicate data from a single database, we selected the study with the larger sample size. Case reports, comments, editorials, letters, and studies not performed on human participants were also excluded. Two researchers (KT and NS) independently searched the literature. After all papers had been assessed, any discrepancies in the responses were identified and discussed to reach a consensus on the best option. Disagreements about the inclusion of a study were resolved through discussion with the senior author (NYF). Data were extracted from each article using a standardized form including the first author, publication year and other information.

### Data extraction

From each study, we extracted information about the publication year, sample size, measures used to assess depression, cutoff point used for each measure, time points for depression assessment, and percent of the prevalence of prenatal or postpartum depression. Publication year, parity, and perinatal depression prevalence were used as continuous variables.

The time of measurement was defined as the first trimester (i.e., 0 to 3 months gestation; Time 1 [T1]), second trimester (i.e., > 3 to 6 months gestation; Time 2 [T2]), third trimester (i.e., > 6 months gestation to childbirth; Time 3 [T3]), 0 to 1 month postpartum (Time 4 [T4]), > 1 to 3 months postpartum (Time 5 [T5]), > 3 to 6 months postpartum (Time 6 [T6]), and > 6 months to 1 year postpartum (Time 7 [T7]). Data from the checkup 1 month after childbirth were extracted separately.

Moreover, for intervention studies, only the baseline data were extracted. For longitudinal studies, only data on the rate of depression from one time point in each period (e.g., prenatal and postpartum) were included in the analyses. For most studies, the first time point was used, as the participants were least familiar with the study tool at that point and were unlikely to exhibit priming effects.

We collected papers that evaluated postpartum depression using the Japanese versions of the EPDS and CES-D.

The EPDS is a self-report instrument measuring postnatal depression with 10 items rated on a 4-point scale (from 0 to 3). The total score ranges from 0 to 30; the higher the score, the worse the symptoms of depression are. The reliability and validity of the Japanese version of the EPDS were reported by Okano, and a cutoff point above 9 was established [16]. Our meta-analysis also included a paper that evaluated depression by using the Japanese version [17] of the CES-D [18]. This tool consists of 20 questions about depression, and the total score ranges from 0 to 60 points. We collected papers that defined the presence of depression based on a CES-D score ≥ 16.

### Statistical analysis

First, we assessed the pooled prevalence of postpartum depression at the time of the checkup 1 month after childbirth. Then, we assessed the pooled prevalence of perinatal or postpartum depression during each period (T1 to T7). Third, we conducted a trend analysis applied the generalized linear mixed model [19]. The *t* tests on the contrast vectors for regression coefficients of the time variable were conducted in order to evaluate the difference between time points in the prenatal period, and the trend of proportion in the post period. Finally, we calculated the relative risk to investigate the differences in the prevalence of postpartum depression between primiparas and multiparas.

We used the *I*^2^ statistic and its 95% CI to estimate heterogeneity. The *I*^2^ statistic was considered high when it was 75% or higher [20]. The significance level was set at *p* < 0.05. The meta-analysis and related statistical analysis were performed with meta-package version 4.9-1 in R version 3.5.0., and the GLIMMIX procedure in SAS ver. 9.4.

## Results

### Search results and included participants

**Fig. 1:**
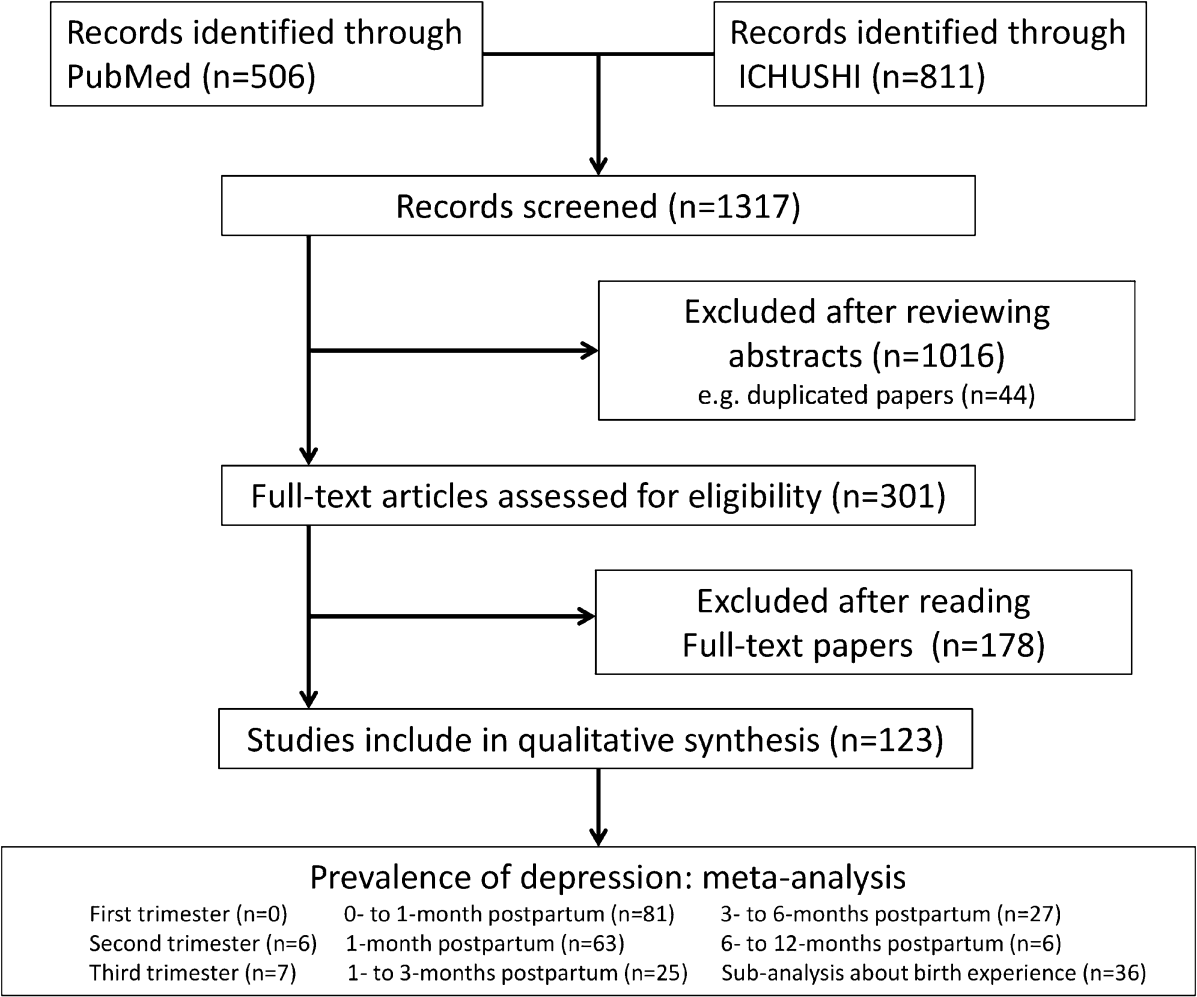
Flowchart of the process of selecting studies for inclusion

After excluding duplicate or irrelevant papers, we found 123 publications that met the inclusion criteria (Fig. 1). The final sample included 108,431 people assessed at the time of the checkup 1 month after childbirth. The sample sizes of the studies ranged from 100 to 82,489 people. More details on the included studies and participants are presented Tables 1 and 2.

**Table 1:** Major characteristics of studies: the prevalence of prenatal and postpartum depression

Author, year	Time classification	Measure	Sample size	Identified cases	Prevalence (%)
Akiyama 2014 [45]	1- to 3-month postpartum	EPDS	936	324	34.6
Amagai 2014 [46]	Third trimester	EPDS	151	33	21.9
Arai 2009 [47]	1-month postpartum	EPDS	149	33	22.1
	0- to 1-month postpartum	EPDS	149	33	22.1
Arakawa 2016 [48]	1-month postpartum	EPDS	257	23	8.9
	0- to 1-month postpartum	EPDS	253	36	14.2
	1- to 3-month postpartum	EPDS	289	26	9.0
Arimoto 2010 [49]	1-month postpartum	EPDS	276	66	23.9
	0- to 1-month postpartum	EPDS	276	66	23.9
Doi 2015 [50]	0- to 1-month postpartum	EPDS	100	20	20.0
Ebine 2007 [51]	1-month postpartum	EPDS	691	131	19.0
	0- to 1-month postpartum	EPDS	691	131	19.0
Emori 2014 [52]	Third trimester	EPDS	110	20	18.2
Fujita 2007 [54]	1-month postpartum	EPDS	1869	222	11.9
	0- to 1-month postpartum	EPDS	1869	222	11.9
Fujita 2015 [53]	1-month postpartum	EPDS	179	38	21.2
	0- to 1-month postpartum	EPDS	179	38	21.2
Fukuda 2011 [55]	1-month postpartum	EPDS	299	57	19.1
	0- to 1-month postpartum	EPDS	299	57	19.1
Fukuzawa 2003 [56]	0- to 1-month postpartum	EPDS	194	25	12.9
	1- to 3-month postpartum	EPDS	194	10	5.2
Fukuzawa 2004 [57]	1-month postpartum	EPDS	664	31	4.7
	0- to 1-month postpartum	EPDS	664	72	10.8
Fukuzawa 2006 [58]	1-month postpartum	EPDS	356	17	4.8
	0- to 1-month postpartum	EPDS	356	32	9.0
Fukuzawa 2011 [59]	1-month postpartum	EPDS	135	8	5.9
	0- to 1-month postpartum	EPDS	135	13	9.6
	1- to 3-month postpartum	EPDS	135	11	8.1
	3- to 6-month postpartum	EPDS	135	13	9.6
	6- to 12-month postpartum	EPDS	135	13	9.6
Goto 2010 [60]	3- to 6-month postpartum	EPDS	378	32	8.5
Hamazaki 2009 [61]	1-month postpartum	EPDS	986	91	9.2
	0- to 1-month postpartum	EPDS	986	91	9.2
Harada 2008 [62]	1-month postpartum	EPDS	820	68	8.3
	0- to 1-month postpartum	EPDS	820	68	8.3
Harada 2009 [63]	1-month postpartum	EPDS	143	18	12.6
	0- to 1-month postpartum	EPDS	143	20	14.0
	3- to 6-month postpartum	EPDS	143	12	8.4
Hashimoto 2014 [64]	1- to 3-month postpartum	EPDS	1222	102	8.3
Honda 2008 [65]	1-month postpartum	EPDS	230	31	13.5
	0- to 1-month postpartum	EPDS	230	31	13.5
Hori 2006 [66]	1- to 3-month postpartum	EPDS	217	29	13.4
Hosoya 2006 [67]	1-month postpartum	EPDS	204	33	16.2
	0- to 1-month postpartum	EPDS	204	33	16.2
Hozumi 2005 [68]	0- to 1-month postpartum	EPDS	110	16	14.5
Ichikawa 2008 [69]	1-month postpartum	EPDS	152	29	19.1
	0- to 1-month postpartum	EPDS	152	29	19.1
Imura 2004 [70]	0- to 1-month postpartum	EPDS	102	21	20.6
Ishii 2010 [71]	1-month postpartum	EPDS	109	4	3.7
	0- to 1-month postpartum	EPDS	109	6	5.5
Ishikawa 2011 [72]	Third trimester	EPDS	424	48	11.3
Iwafuji 2007 [73]	3- to 6-month postpartum	CES-D	129	13	10.1
	6- to 12-month postpartum	CES-D	129	20	15.5
Iwamoto 2010 [74]	1-month postpartum	EPDS	590	30	5.1
	0- to 1-month postpartum	EPDS	590	65	11.0
	3- to 6-month postpartum	EPDS	560	41	7.3
	Third trimester	EPDS	590	101	17.1
Iwata 2016 [75]	0- to 1-month postpartum	EPDS	2854	437	15.3
Iwata 2016 [76]	1- to 3-month postpartum	EPDS	2709	261	9.6
	3- to 6-month postpartum	EPDS	2709	222	8.2
Kanai 2016 [77]	1-month postpartum	EPDS	113	8	7.1
	0- to 1-month postpartum	EPDS	113	8	7.1
Kanazawa 2008 [78]	1-month postpartum	EPDS	111	16	14.4
	0- to 1-month postpartum	EPDS	111	16	14.4
Kaneko 2008 [79]	1-month postpartum	EPDS	103	15	14.6
	0- to 1-month postpartum	EPDS	103	15	14.6
	Second trimester	EPDS	103	13	12.6
Kawai 2017 [80]	0- to 1-month postpartum	EPDS	951	115	12.1
	1- to 3-month postpartum	EPDS	951	47	4.9
	6- to 12-month postpartum	EPDS	951	40	4.2
Kawamura 2006 [81]	0- to 1-month postpartum	EPDS	506	100	19.8
	3- to 6-month postpartum	EPDS	2283	226	9.9
Kikuchi 2010 [83]	1-month postpartum	EPDS	113	17	15.0
	0- to 1-month postpartum	EPDS	113	17	15.0
	3- to 6-month postpartum	EPDS	113	17	15.0
Kinjo 2011 [84]	1-month postpartum	EPDS	152	39	25.7
	0- to 1-month postpartum	EPDS	152	39	25.7
Kinjo 2013 [85]	1- to 3-month postpartum	CES-D	289	96	33.2
	Second trimester	CES-D	320	100	31.3
Kishi 2009 [86]	1-month postpartum	EPDS	160	20	12.5
	0- to 1-month postpartum	EPDS	160	20	12.5
Kobayashi 2017 [10]	1-month postpartum	EPDS	967	191	19.8
	0- to 1-month postpartum	EPDS	967	191	19.8
	3- to 6-month postpartum	EPDS	710	91	12.8
Kondo 2011 [88]	1- to 3-month postpartum	EPDS	129	14	10.9
Kubota 2014 [89]	1-month postpartum	EPDS	690	127	18.4
	0- to 1-month postpartum	EPDS	690	127	18.4
Maruyama 2012 [106]	1-month postpartum	EPDS	143	36	25.2
	0- to 1-month postpartum	EPDS	143	36	25.2
Masuda 2012 [90]	1-month postpartum	EPDS	595	89	15.0
	0- to 1-month postpartum	EPDS	595	89	15.0
Matsukida 2009 [91]	0- to 1-month postpartum	EPDS	1240	102	8.2
Matsuoka 2010 [93]	1- to 3-month postpartum	EPDS	508	69	13.6
Matsuzaki 2009 [94]	1-month postpartum	EPDS	443	40	9.0
	0- to 1-month postpartum	EPDS	436	37	8.5
	1- to 3-month postpartum	EPDS	154	13	8.4
Mishina 2009 [95]	1-month postpartum	EPDS	103	17	16.5
	0- to 1-month postpartum	EPDS	103	17	16.5
Mishina 2010 [96]	1-month postpartum	EPDS	279	43	15.4
	0- to 1-month postpartum	EPDS	279	43	15.4
Mishina 2012 [97]	0- to 1-month postpartum	EPDS	631	87	13.8
	1- to 3-month postpartum	EPDS	1621	182	11.2
	3- to 6-month postpartum	EPDS	312	41	13.1
Mitamura 2008 [98]	1-month postpartum	EPDS	503	40	8.0
	0- to 1-month postpartum	EPDS	503	40	8.0
Miyake 2011[100]	3- to 6-month postpartum	EPDS	771	106	13.7
Miyake 2016 [99]	3- to 6-month postpartum	CES-D	1319	108	8.2
Miyauchi 2014 [102]	1- to 3-month postpartum	EPDS	410	31	7.6
Mori 2017 [104]	1-month postpartum	EPDS	2854	437	15.3
Morikawa 2015 [105]	Second trimester	EPDS	371	68	18.3
Muchanga 2017 [9]	1-month postpartum	EPDS	82,489	11,341	13.7
	0- to 1-month postpartum	EPDS	82,489	11,341	13.7
Murayama 2010 [107]	1- to 3-month postpartum	EPDS	230	23	10.0
Nagatsuru 2006 [108]	1-month postpartum	EPDS	252	62	24.6
	0- to 1-month postpartum	EPDS	252	62	24.6
Nakaita 2012 [109]	1-month postpartum	EPDS	1744	233	13.4
	0- to 1-month postpartum	EPDS	1744	233	13.4
	3- to 6-month postpartum	EPDS	2364	286	12.1
Nakamura 2015 [12]	1-month postpartum	EPDS	215	19	8.8
	0- to 1-month postpartum	EPDS	215	19	8.8
Nakano 2004 [110]	0- to 1-month postpartum	EPDS	169	29	17.2
Ngoma 2012 [111]	1-month postpartum	EPDS	117	7	6.0
	0- to 1-month postpartum	EPDS	117	7	6.0
Nishigori 2015 [113]	0- to 1-month postpartum	EPDS	300	45	15.0
Nishihira 2011 [114]	1-month postpartum	EPDS	179	24	13.4
	0- to 1-month postpartum	EPDS	179	24	13.4
	1- to 3-month postpartum	EPDS	100	13	13.0
Nishikawa 2006 [115]	0- to 1-month postpartum	EPDS	248	37	14.9
	1-month postpartum	EPDS	248	37	14.9
Nishimura 2010 [117]	1-month postpartum	EPDS	178	50	28.1
	0- to 1-month postpartum	EPDS	178	50	28.1
Nishimura 2015 [116]	3- to 6-month postpartum	EPDS	807	83	10.3
Nishioka 2011 [118]	1-month postpartum	EPDS	405	79	19.5
	0- to 1-month postpartum	EPDS	405	79	19.5
	3- to 6-month postpartum	EPDS	653	53	8.1
Nishizono-Maher 2004 [119]	3- to 6-month postpartum	EPDS	693	96	13.9
Ogasawara 2000 [120]	1-month postpartum	EPDS	161	7	4.3
	0- to 1-month postpartum	EPDS	161	7	4.3
Ono 2008 [122]	1-month postpartum	EPDS	151	33	21.9
	0- to 1-month postpartum	EPDS	151	33	21.9
Ono 2009 [122]	3- to 6-month postpartum	EPDS	485	99	20.4
Otake 2014 [123]	Third trimester	EPDS	154	9	5.8
Sadatomi 2011 [124]	1-month postpartum	EPDS	201	19	9.5
	0- to 1-month postpartum	EPDS	201	19	9.5
Sakae 2016 [125]	1-month postpartum	EPDS	110	25	22.7
	0- to 1-month postpartum	EPDS	110	16	14.5
	1- to 3-month postpartum	EPDS	110	17	15.5
	3- to 6-month postpartum	EPDS	110	15	13.6
	6- to 12-month postpartum	EPDS	110	18	16.4
	Third trimester	EPDS	110	19	17.3
Sasaki 2007 [127]	0- to 1-month postpartum	EPDS	314	41	13.1
Sasaki 2012 [126]	1-month postpartum	EPDS	314	49	15.6
	0- to 1-month postpartum	EPDS	314	49	15.6
Sato 2002 [130]	1-month postpartum	EPDS	402	59	14.7
	0- to 1-month postpartum	EPDS	402	84	20.9
Sato 2006 [128]	1-month postpartum	EPDS	189	32	16.9
	0- to 1-month postpartum	EPDS	189	37	19.6
	1- to 3-month postpartum	EPDS	189	27	14.3
	0- to 1-month postpartum	EPDS	103	13	12.6
Sato 2016 [129]	6- to 12-month postpartum	EPDS	677	145	21.4
Satoh 2009 [131]	3- to 6-month postpartum	EPDS	177	42	23.7
Seki 2015 [132]	1- to 3-month postpartum	EPDS	336	71	21.1
Shin 2015 [133]	1- to 3-month postpartum	EPDS	167	18	10.8
Shiraishi 2015 [134]	Second trimester	EPDS	329	19	5.8
Shoji 2009 [135]	1-month postpartum	EPDS	147	7	4.8
	0- to 1-month postpartum	EPDS	147	14	9.5
Suetsugu 2015 [136]	1-month postpartum	EPDS	244	19	7.8
	0- to 1-month postpartum	EPDS	244	19	7.8
Sugimoto 2017 [137]	3- to 6-month postpartum	EPDS	2133	262	12.3
Sugishita 2013 [138]	1-month postpartum	EPDS	121	24	19.8
	0- to 1-month postpartum	EPDS	121	24	19.8
Suzuki 2001 [139]	1-month postpartum	EPDS	1864	275	14.8
	0- to 1-month postpartum	EPDS	1864	275	14.8
Suzuki 2010 [140]	0- to 1-month postpartum	EPDS	684	38	5.6
Suzumiya 2004 [141]	0- to 1-month postpartum	EPDS	780	150	19.2
	1- to 3-month postpartum	EPDS	2334	296	12.7
	3- to 6-month postpartum	EPDS	256	23	9.0
Tachibana 2015 [142]	1-month postpartum	EPDS	1327	169	12.7
	0- to 1-month postpartum	EPDS	1327	169	12.7
	Second trimester	EPDS	1327	128	9.6
Takahashi 2014 [143]	1-month postpartum	EPDS	100	10	10.0
	0- to 1-month postpartum	EPDS	100	10	10.0
Takehara 2009 [144]	3- to 6-month postpartum	EPDS	816	96	11.8
	6- to 12-month postpartum	EPDS	684	65	9.5
Takehara 2018 [145]	1- to 3-month postpartum	EPDS	1305	115	8.8
Tamaki 1997 [149]	1-month postpartum	EPDS	627	114	18.2
	0- to 1-month postpartum	EPDS	627	114	18.2
	1- to 3-month postpartum	EPDS	627	76	12.1
Tamaki 1997 [148]	3- to 6-month postpartum	EPDS	2476	292	11.8
Tamaki 2007 [146]	3- to 6-month postpartum	EPDS	329	61	18.5
Tamaki 2008 [147]	1-month postpartum	EPDS	361	66	18.3
	0- to 1-month postpartum	EPDS	361	66	18.3
Tomari 2012 [150]	0- to 1-month postpartum	EPDS	366	53	14.5
Tomimori 2011 [151]	1-month postpartum	EPDS	271	43	15.9
	0- to 1-month postpartum	EPDS	198	32	16.2
Umezaki 2015 [152]	1-month postpartum	EPDS	114	36	31.6
	0- to 1-month postpartum	EPDS	114	38	33.3
Urayama 2013 [153]	1-month postpartum	EPDS	101	18	17.8
	0- to 1-month postpartum	EPDS	101	33	32.7
Usuda 2016 [154]	1-month postpartum	EPDS	118	11	9.3
	0- to 1-month postpartum	EPDS	118	11	9.3
Usuda 2017 [29]	Second trimester	EPDS	2821	411	14.6
Usui 2013 [155]	1-month postpartum	CES-D	142	41	28.9
	0- to 1-month postpartum	CES-D	142	41	28.9
	Third trimester	CES-D	142	39	27.5
Yamaguchi 2016 [156]	1-month postpartum	EPDS	101	14	13.9
	0- to 1-month postpartum	EPDS	101	20	19.8
	1- to 3-month postpartum	EPDS	101	9	8.9
	3- to 6-month postpartum	EPDS	101	13	12.9
Yamanaka 2012 [157]	1- to 3-month postpartum	EPDS	786	81	10.3
Yamaoka 2016 [158]	3- to 6-month postpartum	EPDS	6534	623	9.5
Yamazaki 2016 [161]	0- to 1-month postpartum	EPDS	363	67	18.5
Yamazaki 2017 [160]	0- to 1-month postpartum	EPDS	105	34	32.4
Yamasaki 2017 [159]	3- to 6-month postpartum	CES-D	399	40	10.0
Yoshida 2017 [162]	0- to 1-month postpartum	EPDS	276	38	13.8

**Table 2:** Major characteristics of studies: the effect of the childbirth experience on postpartum depression

Author, year	Measure	Primiparas		Multiparas	
		Sample size	Identified cases	Sample size	Identified cases
Akiyama 2014 [45]	EPDS	1942	237	1073	87
Arai 2009 [47]	EPDS	73	21	76	12
Doi 2015 [50]	EPDS	43	8	50	7
Fukuda 2011 [55]	EPDS	131	28	168	29
Fukuzawa 2004 [57]	EPDS	383	58	281	14
Fukuzawa 2006 [58]	EPDS	188	18	168	11
Fukuzawa 2011 [59]	EPDS	73	11	62	2
Hamazaki 2009 [61]	EPDS	443	56	543	35
Hozumi 2005 [68]	EPDS	49	13	61	3
Ichikawa 2008 [69]	EPDS	68	17	84	12
Ishii 2010 [71]	EPDS	64	4	45	2
Kanai 2016 [77]	EPDS	72	7	41	1
Kanazawa 2008 [78]	EPDS	42	6	69	10
Kikuchi 2007 [82]	EPDS	192	34	235	25
Kishi 2009 [86]	EPDS	83	12	77	8
Kishimoto 2013 [87]	EPDS	99	14	121	9
Kobayashi 2017 [10]	EPDS	598	155	369	36
Matsumoto 2011 [92]	EPDS	332	62	343	38
Mishina 2010 [96]	EPDS	164	34	115	9
Mitamura 2008 [98]	EPDS	211	20	312	20
Miyamoto 2012 [101]	EPDS	72	13	56	6
Mori 2016 [103]	EPDS	1808	430	1597	161
Muchanga 2017 [9]	EPDS	24,340	4276	57,351	6907
Nagatsuru 2006 [108]	EPDS	137	42	115	20
Nakamura 2015 [12]	EPDS	114	12	101	7
Nakano 2004 [110]	EPDS	75	15	85	9
Ngoma 2012 [111]	EPDS	41	5	76	2
Ninagawa 2005 [112]	EPDS	177	30	159	17
Ono 2008 [121]	EPDS	85	23	66	10
Sato 2002 [130]	EPDS	215	48	187	36
Satoh 2009 [131]	EPDS	99	29	78	13
Takehara 2018 [145]	EPDS	721	122	585	52
Tamaki 1997a [149]	EPDS	353	77	273	36
Tamaki 1997b [148]	EPDS	1034	147	1437	144
Tomari 2012 [150]	EPDS	136	38	177	15
Urayama 2013 [153]	EPDS	82	30	19	3
Watanabe 2008 [11]	EPDS	111	15	124	15
Yamaguchi 2016 [156]	EPDS	45	12	56	8
Yoshida 2017 [162]	EPDS	128	23	148	15

### Prevalence of perinatal depression and subgroup analysis

**Fig. 2:**
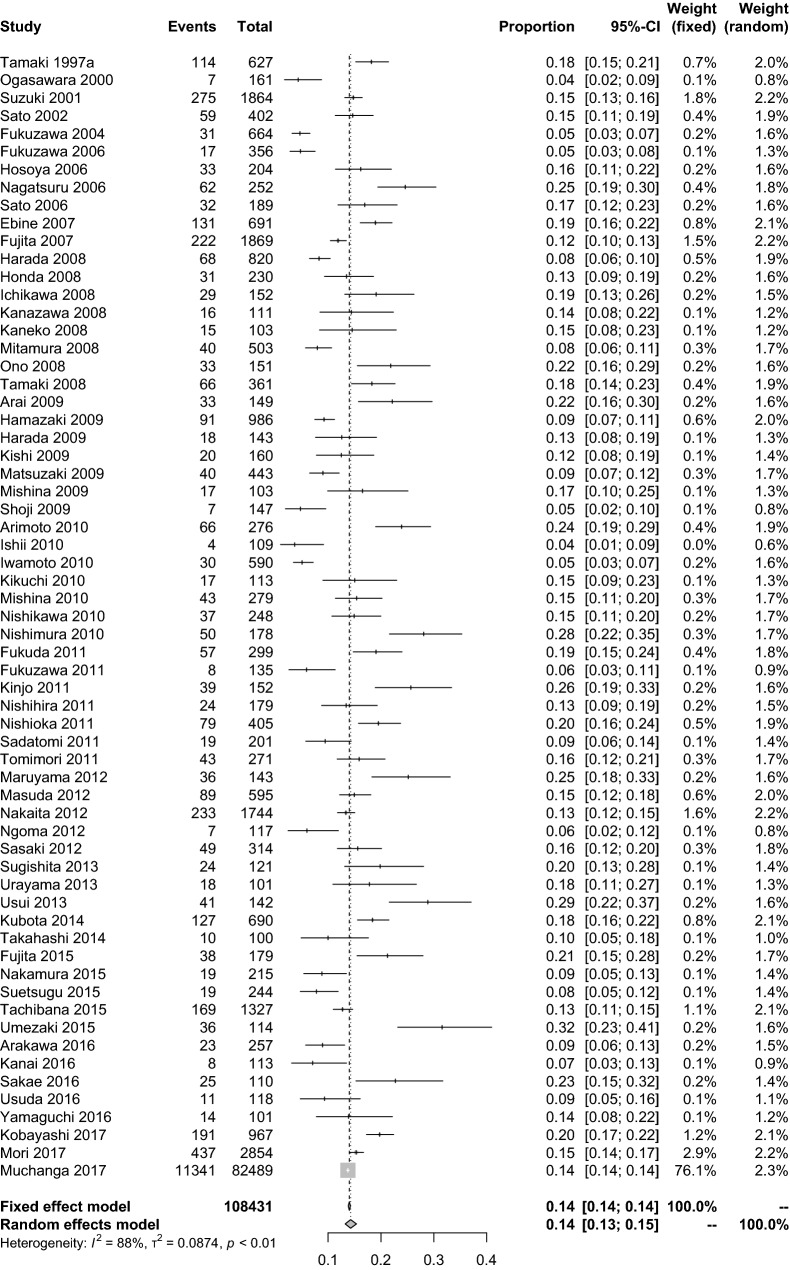
Prevalence of postpartum depression 1 month after childbirth

The point prevalence of postpartum depression at 1 month after childbirth was calculated by integrating the 108,431 people from 63 publications and was found to be 14.3%. (95% CI 13.2–15.4%). The level of heterogeneity was *I*^2^ = 88.3%. Because of the high heterogeneity, the prevalence was calculated by a random-effects model (Fig. 2).

**Fig. 3:**
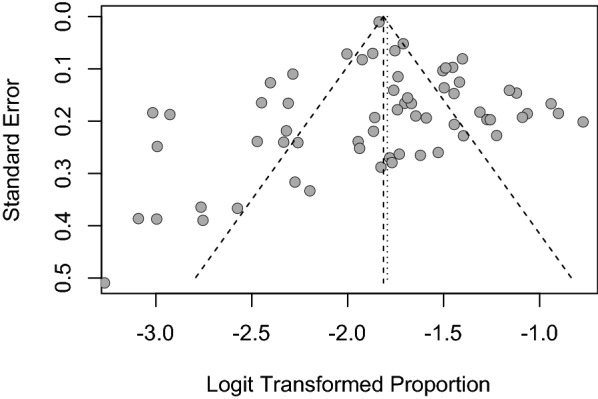
Funnel plot at 1 month after childbirth

A visual inspection of the funnel plot at 1 month after childbirth revealed symmetry (Fig. 3), and Egger’s regression test for funnel plot asymmetry was statistically nonsignificant (*t* = 0.5958, *p* = 0.5535).

**Fig. 4:**
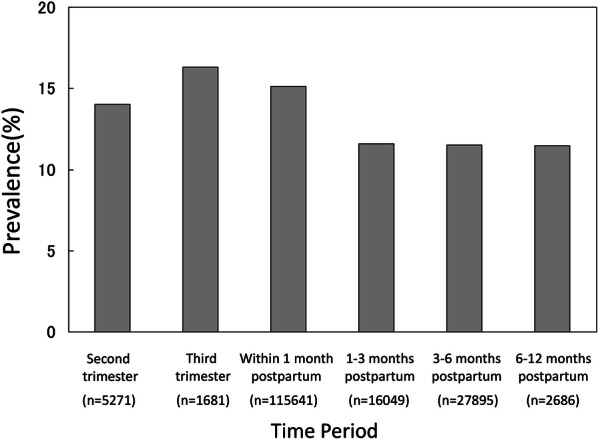
Prevalence of perinatal depression as a function of the time period

The period prevalence of depression at T1 could not be calculated due to a lack of reported data. The period prevalence of depression at T2 was 14.0% (95% CI 9.4–20.3%) based on the inclusion of 5271 people from 6 papers. Similarly, the period prevalence of depression was 16.3% at T3 (95% CI 12.2–21.5%), 15.1% at T4 (95% CI 14.2–16.1%), 11.6% at T5 (95% CI 9.2–14.5%), 11.5% at T6 (95% CI 10.4–12.7%) and 11.5% at T7 (95% CI 6.5–19.5%). From T2 to T7, high heterogeneity was observed in the prevalence data for all periods, so the prevalence was calculated by using a random-effects model (Fig. 4).

**Fig. 5:**
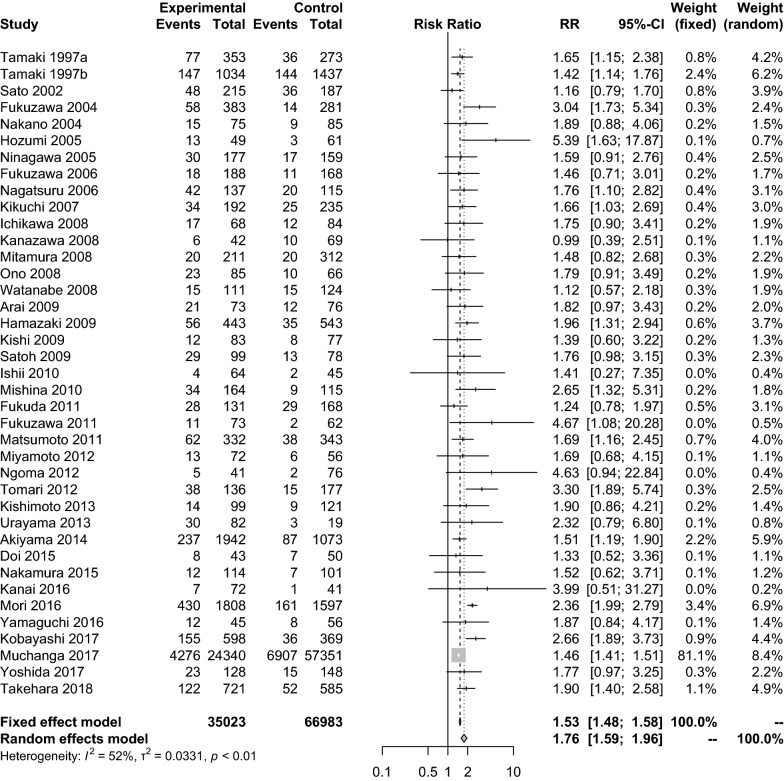
Relative risk of the prevalence of postpartum depression between primiparas and multiparas

Next, a subanalysis of the effect of the childbirth experience on postpartum depression was performed. The data for a total of 102,006 people described in 39 papers were integrated, and a meta-analysis was performed at the relative risk level. The result showed that primiparas had a significantly higher prevalence of postpartum depression than multiparas, with a relative risk of 1.76 (95% CI 1.59–1.96). The level of heterogeneity was *I*^2^ = 52.2%; the meta-analysis of relative risk was performed using a random-effects model (Fig. 5).

### Trend analysis of the prevalence of perinatal depression

We performed a trend analysis by applying the generalized linear mixed model where outcome was existence of depression; link function was logit function; fixed effects were time (6 time points from T2 to T7; nominal variable) and scale (CES-D or EPDS); random effect was trial. As a result of the F test, there was no statistically significant difference between CES-D and EPDS in the prevalence of perinatal depression (*F* = 0.46, *p* = 0.501). The t tests on the contrast vectors for regression coefficients of the time variable were conducted in order to evaluate the difference between two time points in the prenatal period, and the trend of proportion in the postpartum period. The contrast vector for prenatal period was set as (− 1, 1, 0, 0, 0, 0), and postpartum period was set as (0, 0, 3, 1, − 1, − 3). The contrast vector for prenatal–postpartum comparison was set as (2, 2, − 1, − 1, − 1, − 1). As a result of trend analysis, a prevalence of prenatal depression increased statistically significantly over time (*t* = 3.78, *p* = 0.001), and a prevalence of postpartum depression decreased statistically significantly over time (*t* = 6.00, *p* < 0.001). Comparing the prevalence of prenatal and postpartum depression, prevalence of prenatal depression was statistically significantly higher than that of postpartum depression (*t* = 4.11, *p* < 0.001).

### Sensitivity analysis of the prevalence of perinatal depression

Additionally, a sensitivity analysis was performed to examine the robustness of the data. In particular, the analysis focused on heterogeneity. We found that when the study with the largest sample size (*n* = 82,489), i.e., the JECS [9], was excluded, the prevalence of depression was 14.1% at 1 month postpartum (95% CI 12.8–15.5%, *I*^2^ = 88.1%, *n* = 25,942). There was no statistically significant difference in the prevalence of depression with or without the JECS data, and the heterogeneity was the same with or without JECS data.

The EPDS is the most frequently used measure to evaluate perinatal depression in women worldwide [21], so we examined the prevalence of perinatal depression only with statistical data from the EPDS. The prevalence of perinatal depression after the sensitivity analysis is presented below.

The point prevalence of postpartum depression at 1 month after childbirth with the CES-D data excluded was 14.1%. (95% CI 13.1–15.2%). The level of heterogeneity was *I*^2^ = 88.0%.

The period prevalence of depression at T1 could not be calculated due to a lack of reported data. The period prevalence of depression at T2 was 11.8% (95% CI 8.6–15.9%). Similarly, the period prevalence of depression was 14.9% at T3 (95% CI 11.1–20.0%), 15.0% at T4 (95% CI 14.1–15.9%), 11.0% at T5 (95% CI 8.8–13.7%), 11.8% at T6 (95% CI 10.6–13.1%), and 10.8% at T7 (95% CI 5.5–20.1%). There was little statistical influence of the CES-D data on the robustness of the data.

## Discussion

Our study is the first to use a meta-analysis to investigate the reliable prevalence of perinatal depression among Japanese women. The most important finding is that the point prevalence of postpartum depression was 14.3% 1 month after childbirth. The JECS [9] is a large-scale study compared with other studies, so we tried to reanalyze the data with the JECS data excluded. The prevalence of postpartum depression and heterogeneity 1 month after childbirth were almost the same with or without the JECS data. While the JECS already identified the reliable prevalence of postpartum depression, our research confirms the extent of the heterogeneity in postpartum depression among Japanese women.

According to the DSM-IV-TR [22], maternity blues are defined as depressive episodes that develop by the fifth day after childbirth and then disappear within 2 weeks. It is recommended that maternity blues and postpartum depression be clearly distinguished [22]. Thus, it might be important to establish a sampling time to investigate the condition of postpartum depression 1 month after childbirth to exclude the possibility of maternity blues.

In Japan, the rate of infant health checkups 1 month after childbirth is high at 83.6% [23], and infants’ mothers are also checked for health problems at that time. Since Okano created the Japanese version of the EPDS [16], this screening tool has been used for the early detection of a high risk of depression in mothers. Epidemiological studies of perinatal depression are mainly conducted by public health nurses and midwives in Japan. Although they often report research results in Japanese, sampling bias is less likely in these studies.

In addition, every year, approximately 100 women commit suicide in Japan because of worry about childcare, and the number has remained high [24]. Recently, Takeda analyzed the abnormal deaths of perinatal women in Tokyo from 2005 to 2014 and reported that 63 suicides occurred during this period (23 cases during pregnancy and 40 cases under 1 year postpartum) [25]. These women were suffering from mental illnesses, such as depression, and this figure was more than double the maternal mortality rate due to obstetric abnormalities. Therefore, it is important to estimate the prevalence of postpartum depression in Japan. In addition, postpartum depression may lead to child abuse [26]. Therefore, to protect the health of children, more substantial measures against perinatal depression are needed.

Furthermore, the prevalence of postpartum depression in primiparas is higher than that in multiparas. This is a fundamentally important finding that has major implications for the national health care plan in Japan. There may be several reasons for this result. First, multiparas are expected to have some experience adapting to the stress of childbirth and childcare through the pregnancy experience. Second, a woman with a history of postpartum depression is known to have a high risk of depression during the birth of her second child [27]. For this reason, a high-risk multipara has already received psychological education for perinatal depression and may take preventive measures. Third, if a woman suffered from perinatal depression in her first childbirth and did not receive adequate care, her motivation to give birth to a second child may be reduced. Further research is needed to provide details on the relationship between postpartum depression and family planning.

According to the DSM-5 [28], 50% of cases of postpartum depression are known to have developed during pregnancy. Therefore, mood disorders not only postpartum, but also during pregnancy have also been attracting attention. Interestingly, the prevalence of depression increases as childbirth approaches during pregnancy and the prevalence decreases over time in the postpartum period. In particular, the prevalence of depression was the highest in the third trimester of pregnancy; however, a previous report suggested using different cutoff values for the EPDS for the periods before and after pregnancy [29]. A similar trend has been observed in the United States, and large-scale cohort studies have reported that the prevalence of perinatal depression reaches its peak just before childbirth [30]. During pregnancy, the prevalence of depression increases as childbirth approaches.

Sleep disorders, such as restless leg syndrome and frequent awakening at night, are known to occur most often in the third trimester of pregnancy [31, 32]. On the other hand, sleep quality improves over time after childbirth [33]. In addition, urinary incontinence may also raise the risk of perinatal depression [34]. During pregnancy, frequent urination is common [35], and the degree of urinary incontinence is reported to increase as childbirth approaches [36]. The worsening of frequent urination may affect the prevalence of depression during pregnancy. These studies attributed the increase in prevalence to organic problems of an epidemiological nature, but it is not possible to claim direct causal links between depression and biological factors.

The cessation of the use of antidepressants during pregnancy may also affect the increase in maternal depression prevalence. Pearlstein reported that although antidepressants are the most common treatment for postpartum depression, women tend to prefer psychotherapy [37]. Certainly, there is strong evidence for the effectiveness of structured psychotherapy, such as cognitive behavioral therapy (CBT) [38], interpersonal psychotherapy (IPT) [39] and psychological education [30, 40], for treating and preventing perinatal depression. Therefore, psychotherapy should be considered the first choice, depending on the patient’s condition. However, the need for drug therapy should also be considered. Suzuki reported that depression declined in Japanese women who had been treated for depression after they had stopped antidepressants after pregnancy [41]. The JECS also showed that Japanese women tend to refrain from taking drugs when pregnant. These women again increase their rate of medication after birth [42]. Interestingly, the incidence of postpartum depression is reported to be very low among women with no history of mental illness [27]. In other words, patients with postpartum depression may have had a predisposition for depression before onset. It was also reported that women who discontinued antidepressant medication experienced a relapse of major depression during pregnancy significantly more frequently than women who maintained their medication (hazard ratio, 5.0; 95% confidence interval, 2.8–9.1; *p* < 0.001) [43]. Therefore, drug treatment strategies should be carefully assessed by a psychiatrist with a case-by-case approach when pharmacotherapy is administered to perinatal women.

## Limitations

This study has several limitations. First, the prevalence of depression in the perinatal period was reported based on screening test results. This approach may have resulted in the inclusion of people who should not be clinically diagnosed with depression, such as people with bipolar affective disorder. We included studies that used the CES-D and EPDS as tools to evaluate depression. Although other depression screening methods, such as the 2-item method, the Self-Rating Depression Scale (SDS), the Research Diagnostic Criteria (RDC) and the Mini International Neuropsychiatric Interview (MINI), have been reported, the EPDS and CES-D are the major tools for evaluating a depressed state during the perinatal period according to a prior study [44]. Because group heterogeneity increases when another evaluation scale is added, we limited our analysis to those two tools. Second, a recent report suggested that the cutoff should be 12 rather than 9 points when using the Japanese version of the EPDS to screen for depression during pregnancy [29]. It is possible that the prenatal and postpartum scores should not be assessed in the same way. Third, an internal bias may have been present, because our meta-analysis included only Japanese patients.

## Conclusion

Our meta-analysis provided reliable estimates of the prevalence of perinatal depression among Japanese women. The point prevalence of postpartum depression 1 month after childbirth was found to be 14.3%, and the data had high heterogeneity. Our results indicated that during pregnancy, the prevalence of depression increased as childbirth approached, and the prevalence decreases over time in the postpartum period. In addition, we found that the prevalence of postpartum depression in primiparas was higher than that in multiparas. Hence, we suggest that healthcare professionals need to pay more attention to primiparas than multiparas regarding postpartum depression.

## Data Availability

All data generated or analyzed during this study are included in this published article.
